# Incidentally detected renal leiomyosarcoma with inferior vena cava tumor thrombus: a case report with review of the literature

**DOI:** 10.3389/fsurg.2025.1640444

**Published:** 2025-08-21

**Authors:** Puze Wang, Jinze Li, Qian Liu, Dong Lv, Liangren Liu

**Affiliations:** ^1^ Urology Department, People’s Hospital of Deyang City, Deyang, Sichuan, China; ^2^ Pathological Department, People’s Hospital of Deyang City, Deyang, Sichuan, China; ^3^ Urology Department, West China Hospital, Sichuan University, Chengdu, Sichuan, China

**Keywords:** renal leiomyosarcoma, case report, inferior vena cava tumor thrombus, review, comprehensive treatment

## Abstract

**Background:** Kidney cancer is the 14th most common cancer worldwide. On the basis of the histological characteristics of kidney cancers, most kidney cancers are renal cell carcinomas. Renal leiomyosarcoma (LMS) is extremely rare and malignant and accounts for less than 1% of all kidney cancer cases. Our study aims to gain deeper insight into the pathological characteristics and synthesized treatment of this uncommon type. Thus, we conducted a retrospective analysis of one patient who was diagnosed with renal leiomyosarcoma and underwent comprehensive treatment at the People’s hospital of Deyang City.

## 1 Introduction

According to data from the Department of United States Cancer Statistics, kidney cancer has already become the third most common carcinoma of urinary cancers. In 2021, nearly 70,000 new renal-derived cancer cases were reported around the USA (1). Histologically, approximately ninety percent of kidney tumors are renal cell carcinomas (RCCs). Other remaining subtypes, including adenocarcinoma and squamous cell carcinoma, account for less than ten percent of kidney cancers (2). Renal leiomyosarcoma (LMS), first reported in 1,967, is classified as a rare and aggressive mesenchymal tumor that accounts for nearly 0.1% of all renal malignancies and is associated with early invasion and metastasis (3, 4). The published literature reveals that LMS is likely to occur in females with a mean age of 50–60 years (5). Overall, LMS is always correlated with a poor prognosis because of its high tendency for local recurrence and hematogenous spread (6).

## 2 Patient and observation

### 2.1 Patient characteristics

A 57-year-old male presented to our hospital with slight pain in the right renal region for more than one year, without other accompanying symptoms such as hematuria, fever or radiating pain. The male did not seek medical attention immediately because the pain was not severe and could be relieved spontaneously after taking nonsteroidal anti-inflammatory drugs (NSAIDs). Approximately one month earlier, the pain worsened, and a palpable firm mass developed under the subhepatic region. The patient was eventually diagnosed with a “right renal tumor” and admitted to our urology department.

### 2.2 Clinical findings

A painless 10–15 cm mass was palpable in his right subcostal margin with a local projection of the skin upon clinical examination, accompanied by percussion pain in the right kidney region. Other abnormalities were not revealed.

### 2.3 Imaging examination

**Figure 1:**
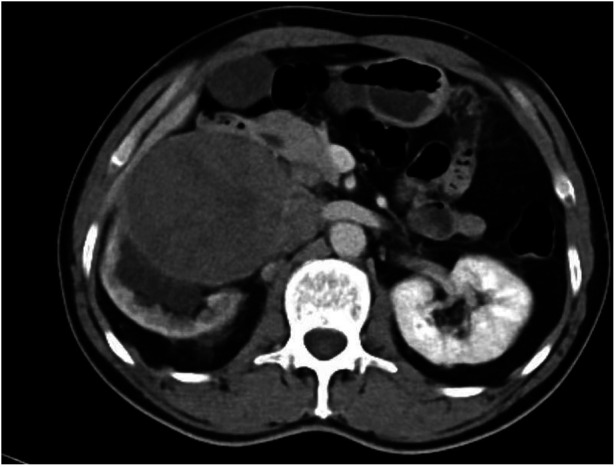
The enhanced scan demonstrated delayed enhancement for the tumor.

**Figure 2:**
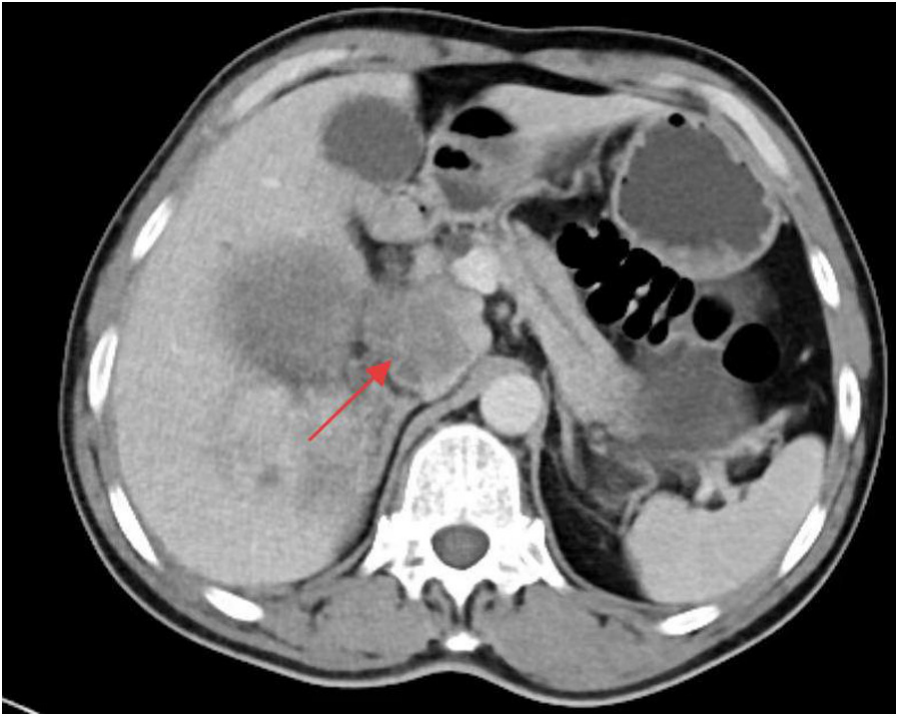
The tumor embolus in the inferior vena cava.

**Figure 3:**
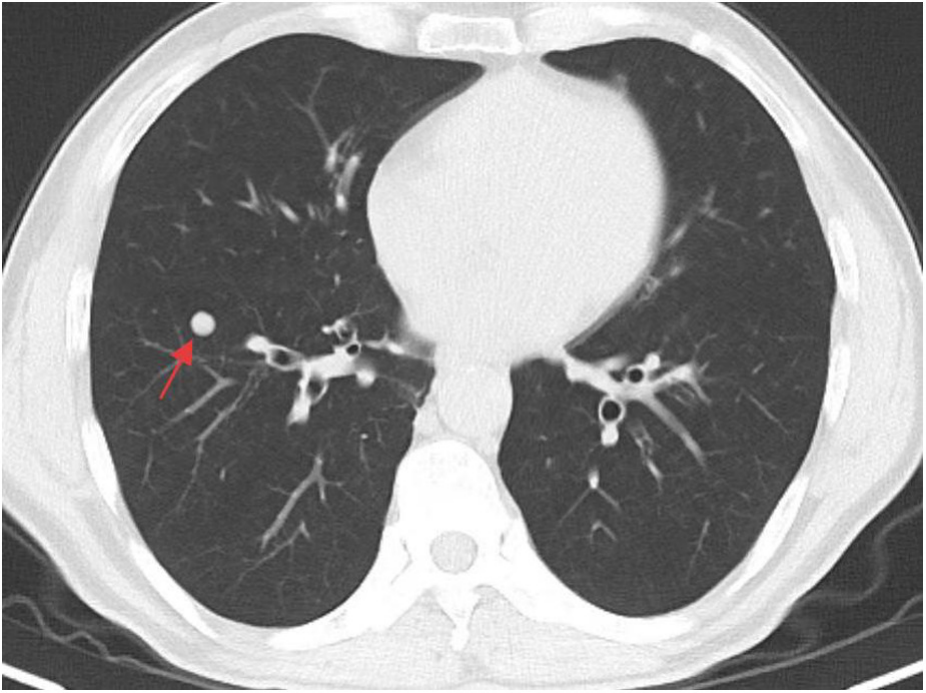
The metastatic tumors in the lung.

A whole-abdominal plain CT scan revealed a tumor with an abundant blood supply located in the region near the caudate lobe and right kidney. The enhanced scan demonstrated delayed enhancement (Figure 1). A tumor embolus was found in the inferior vena cava (Figure 2). In addition, perioperative renal dynamic imaging and glomerular filtration rate (GFR) measurements revealed decreased blood perfusion and glomerular filtration function in the right kidney. Moreover, a chest CT scan revealed scattered solid nodules in both lungs, which were suspected to be metastatic tumors (Figure 3).

### 2.4 Therapeutic intervention

**Figure 4:**
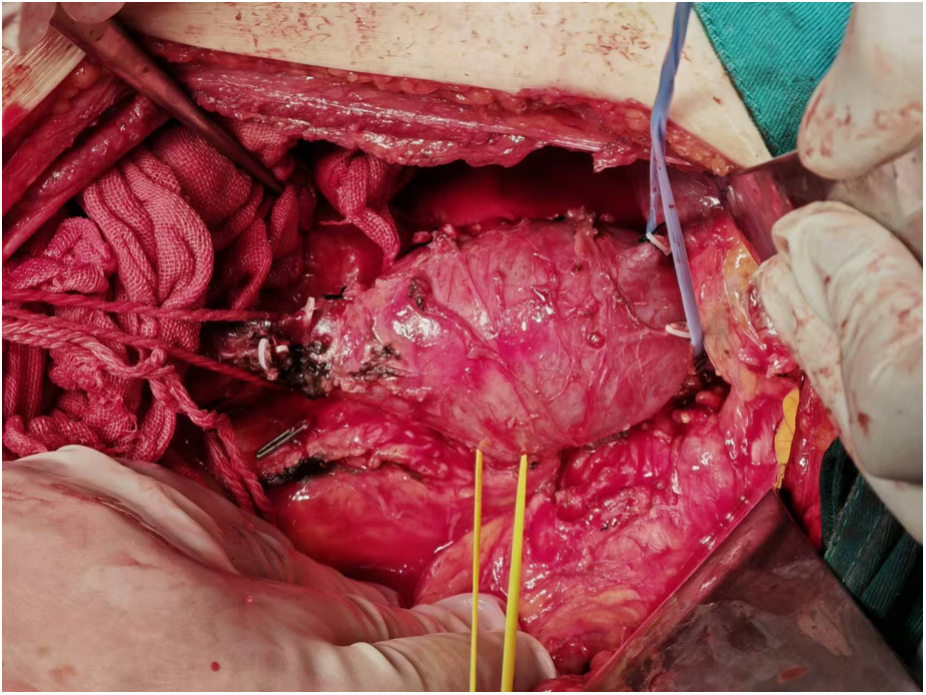
Intraoperative findings revealed caval tumor thrombus.

To reduce cancer-related complications, the patient underwent laparoscopic radical resection of the right renal cancer under general anesthesia. Laparoscopic radical nephrectomy was forced to be converted to open radical nephrectomy because of severe adhesions. In addition, the tumor embolus in the inferior vena cava was resected as much as possible (Figure 4). Local abdominal lymph nodes were also dissected. All the removed samples were routinely sent for pathological examination.

### 2.5 Follow-up

**Figure 5:**
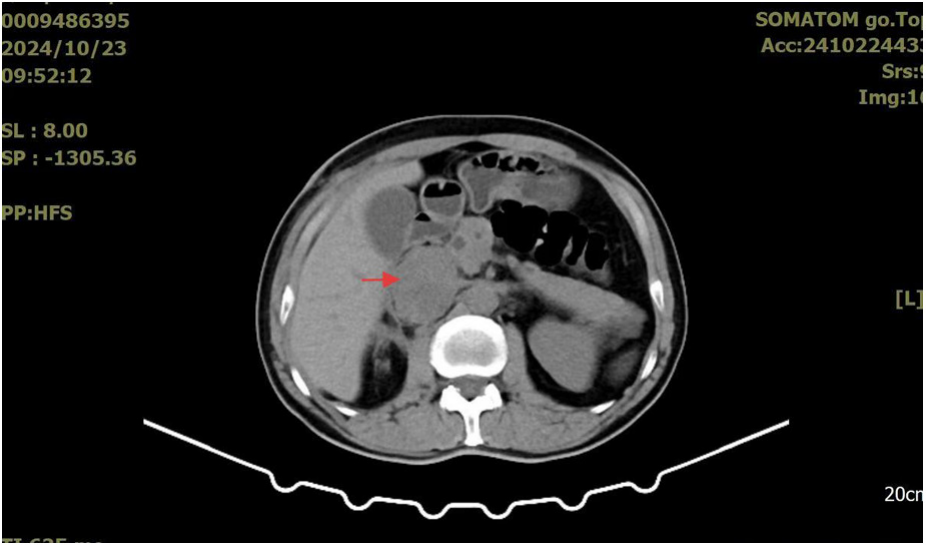
Postoperative CT scan reveals mass around inferior vena cava.

**Figure 6:**
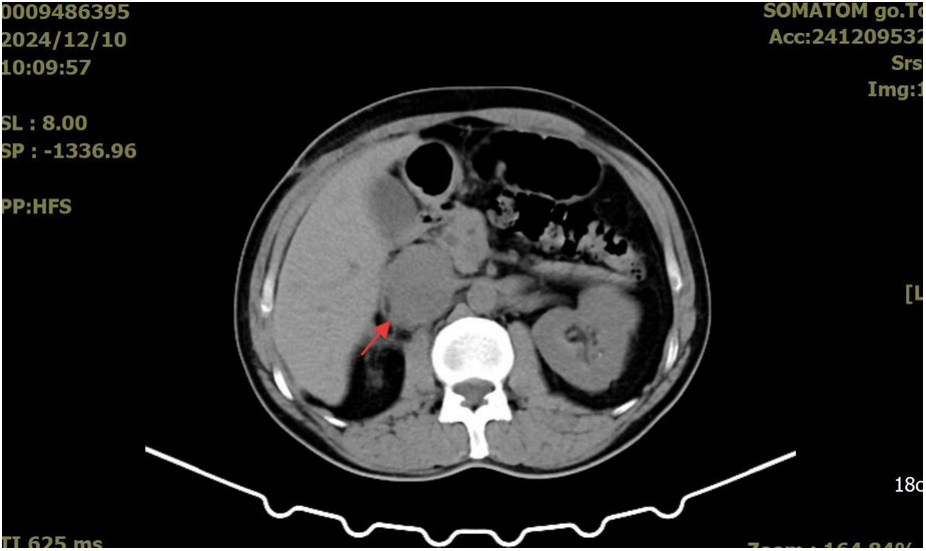
The mass around inferior vena cava become larger.

The patient returned to the oncology center for postoperative follow-up one month later. An abdominal computed tomography (CT) scan revealed a 3.5*3.0 cm low-density mass around and within the inferior vena cava (Figure 5). He received adjuvant chemotherapy every three weeks with ifosfamide (2,400 mg, days 1–4) and epirubicin (120 mg, day 1). Unfortunately, three months after the last abdominal CT scan, a larger mass (5.1*4.2 cm) was detected (Figure 6). In addition, no significant changes were found in the metastatic lesions in the lungs of the patient.

### 2.6 Patient's perspective

During the diagnostic process, the patient was informed of all diagnostic possibilities and prognoses depending on the ancillary test results. Moreover, all possible situations of surgical and chemical intervention were also reported before our treatment. To date, all the doubts of this patient were resolved during his follow-up period.

### 2.7 Pathological results

**Figure 7:**
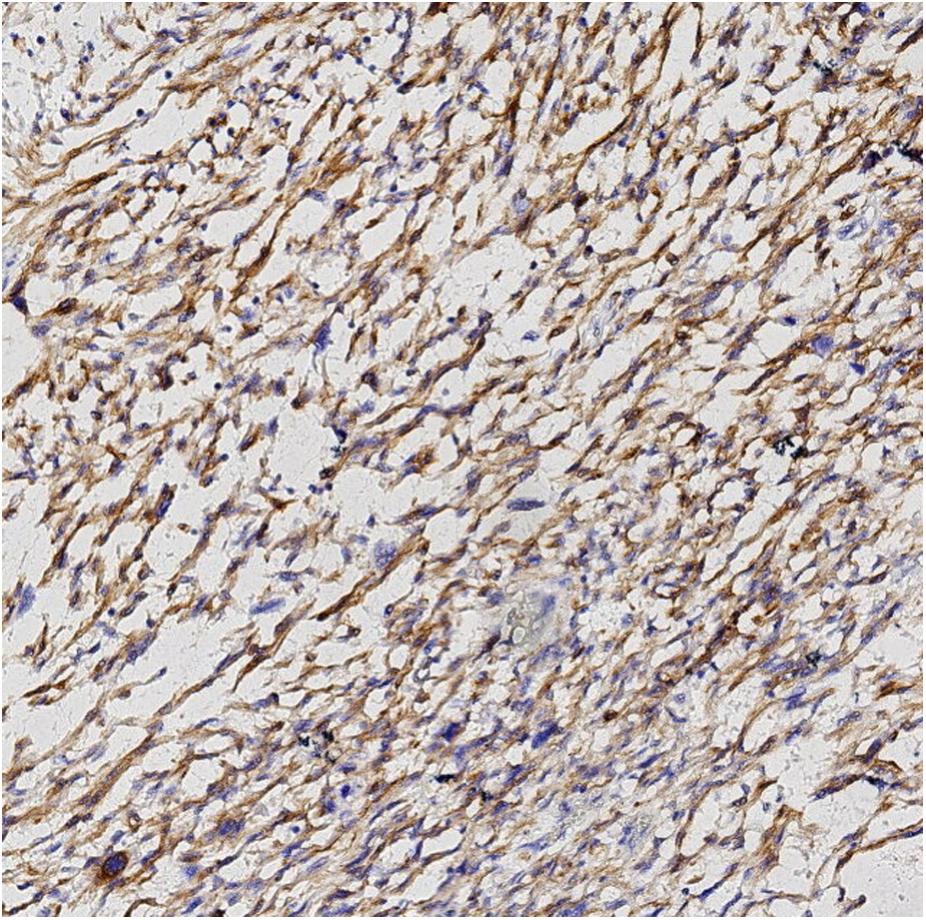
Caldesmon immunohistochemical staining.

**Figure 8:**
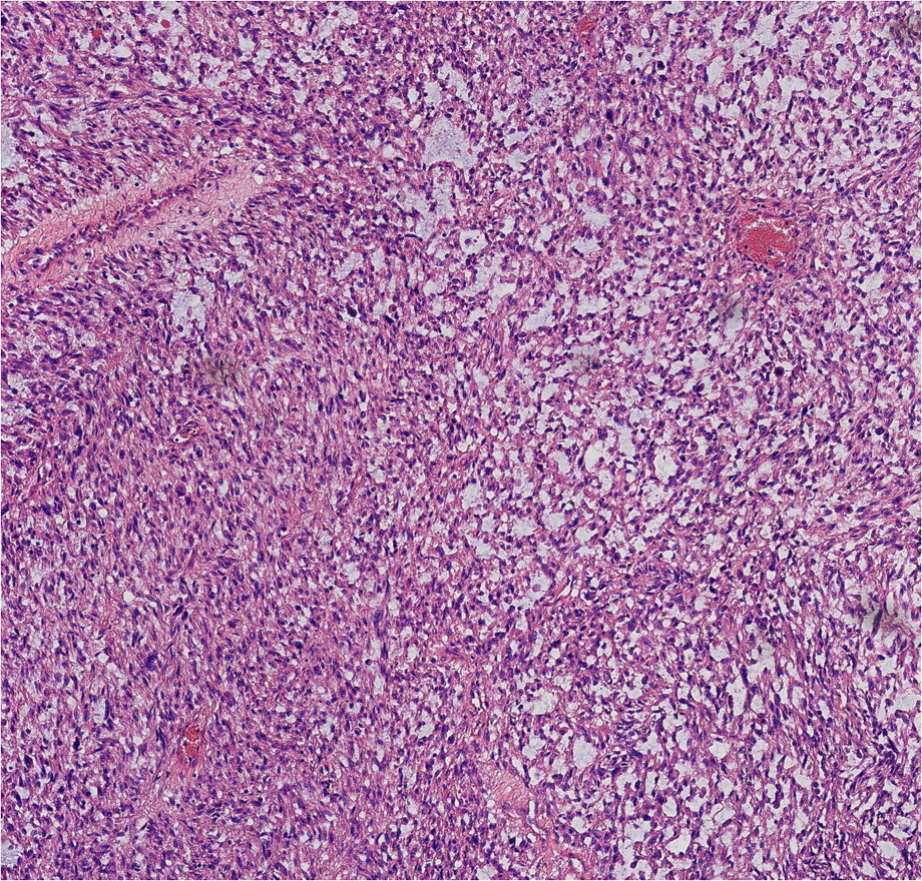
Typical microscopic morphology of LMS (HE staining, 10x).

**Figure 9:**
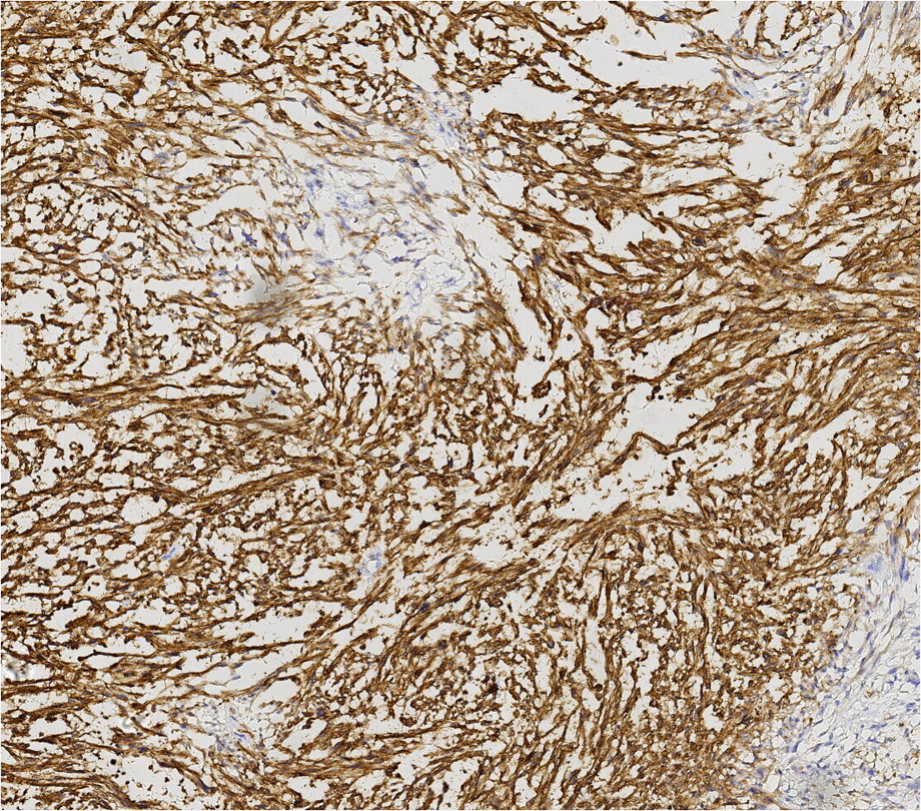
Desmin immunohistochemical staining.

Finally, pathological results confirmed that the tumor was renal LMS. According to the pathological result of Hematoxylin and Eosin (HE) stainning, renal LMS performed a significant cellular pleomorphism with foci of tumor necrosis. In addition, most of the composition in the field of view consists of spindle cells, combined with smooth muscle bundles. Immunohistochemical staining revealed varying degrees of positivity for several markers, including Desmin, EMA, Caldemson, SMA, SMARCA4/Brgl and CA IX. Other non-muscle origin markers like HMB-45 and S-100 were not observed (Figures 7–9).

## 3 Discussion

LMS are malignant neoplasms that arise from the mesenchyme in the smooth muscle of various organs and account for approximately 10%–20% of all types of soft tissue sarcomas (STSs) (7). Renal LMS is likely to arise from intrarenal blood vessels and the renal pelvis (5). According to etiologic research, few predisposing factors for renal LMS have been recognized to date (8). Moreover, compared with other commonly diagnosed solid tumors, almost all categories of LMS exhibit a lower sensitivity, with a less than 30% overall rate of medical therapeutic reactions (9).

Currently, among all kinds of screening tools, magnetic resonance imagingmagnetic (MRI) may have the greatest potential in diagnosing LMS. According to the published literature, low apparent diffusion coefficient (ADC) values, irregular margins and intermediate/hyperintensity in T2-weighted images may be common characteristics of LMS (10). However, only histopathological examination could be the gold standard for diagnosing renal LMS. The characteristics of renal LMS are consistent with those of other locations, which exhibit cellular pleomorphism accompanied by foci of tumor necrosis. The typical microscopic features are characterized by an interlacing pattern of spindle cells and smooth muscle cells. In addition, immunohistochemistry results always reveal positive markers of muscle origin, which is consistent with the findings in our case (5).

At present, the most useful treatment for early renal LMS is surgical intervention with negative margins. Owing to the high local recurrence rate of LMS, radical nephrectomy should first be considered rather than simple partial nephrectomy unless the renal LMS has been identified as low grade through pathological examination (11). For primary renal LMS with a large tumor size, local invasive metastasis, or medium- or high-pathologic grade, radical or extended radical nephrectomy combined with perioperative and postoperative neoadjuvant treatments (including radiotherapy and chemotherapy) can be selected, which needs coordination by the surgeon and the radiation oncologist. Published studies have shown that R0 or R1 resection after neoadjuvant radiotherapy in patients diagnosed with intermediate/high-grade retroperitoneal soft tissue sarcoma (RSTS) can lead to favorable 5-year survival (12). Other studies revealed that for retroperitoneal/intra-abdominal STS where local recurrence can cause undue morbidity, radiotherapy (RT), including intensity-modulated RT and protons, might improve the comprehensive therapeutic effect. However, according to data from the STRASS trial (one of the randomized studies that evaluated neoadjuvant radiotherapy for retroperitoneal STS), whether neoadjuvant radiotherapy could become a standard application for STS treatment is still controversial (13, 14). However, although an updated meta-analysis involving 1,953 participants revealed that adjuvant chemotherapy has advantages in decreasing the overall recurrence rate of overall resectable localized STS (15), available comprehensive evaluations of neoadjuvant therapy for simple resectable renal LMS are still lacking. Neoadjuvant chemotherapy with doxorubicin and dacarbazine may have a positive effect on retroperitoneal LMS according to an unfinished prospective randomized controlled trial, but the final analysis of prognostic outcomes is still ongoing (16). In addition, chemotherapy with single agents or anthracycline-based combination regimens has been widely used among patients with unresectable or metastatic STS (17). For various histologic subtypes of unresectable or metastatic STS, gemcitabine in combination with docetaxel, vinorelbine, or dacarbazine has been confirmed to be effective. In addition, a new DNA-binding agent, trabectedin, has been shown to lead to greater progression-free survival than dacarbazine in the treatment of patients with LMS (18, 19). Moreover, according to the literature, targeted therapy could also delay the progression of LMS. A multitarget tyrosine kinase inhibitor, pazopanib, has already been demonstrated to be effective in prolonging the median progression-free survival (PFS) of patients with multiple advanced STS subtypes (20–22). However, the overall prognosis of advanced STS patients is still poor (17). Owing to the limited sample size, the comprehensive efficacy of renal LMS treatment still needs long-term research.

The progression and prognosis of renal LMS have been demonstrated to be associated with genetic factors. Studies have reported that the expression of the p16, p53, MED12, PRUNE2 and c-Myc tumor suppressor proteins might hold potential value in the prognostic assessment of renal LMS (23–25). In addition, a recent study revealed that TRPV4 may significantly promote the progression of leiomyosarcoma. TRPV4 is a nonselective cation channel that allows the passage of Ca^2+^. It contributes to the progression of LMS by directly stimulating the FAK pathway and indirectly activating the FAK/PI3K/AKT/GSK3β signaling pathway by promoting ECM1 expression and secretion, which may provide new ideas and targets for the clinical treatment of renal LMS in the future (26).

Currently, the diagnosis of various types of renal tumors primarily relies on imaging evaluations, such as contrast-enhanced CT and renal MRI. Totally, it is difficult for surgeons to distinguish between RCCs and renal LMS based solely on imaging examinations. Possible features that differentiate renal LMS from RCCs on CT scans are that LMS may expand to large sizes without lymphadenopathy, and tumor calcification rarely occurs (27). Moreover, the typical contrast-enhanced CT feature of RCCs demonstrates a “fast-in and fast-outfast-in and fast-out” pattern, which performs a marked enhancement in the arterial phase and a rapid washout in the delayed phase. By contrast, due to the less tumor vasculature, LMS may demonstrates a different venous phase with gradual enhancement of contrast agent (28, 29). Unfortunately, specific imaging characteristics has not been observed in renal LMS due to its rarity (6). Thus, image-guided percutaneous renal mass biopsy is still a significant and safe perioperative tool for renal LMS diagnosing (30). In addition, intraoperative frozen section examination may also guide the selection of surgical approaches.

## 4 Conclusion

Renal LMS is extremely rare and lethal. To date, the most effective treatment remains radical surgical intervention. Even if LMS has metastasized locally or to distant sites, although radiotherapy, chemotherapy or targeted therapies may delay its progression, the overall prognosis remains unfavorable. Thus, further research on renal LMS is warranted in the future.

## Data Availability

The raw data supporting the conclusions of this article will be made available by the authors, without undue reservation.
